# Polyphasic characterization of carbapenem-resistant *Klebsiella pneumoniae* clinical isolates suggests vertical transmission of the *bla*_KPC-3_ gene

**DOI:** 10.1371/journal.pone.0247058

**Published:** 2021-02-26

**Authors:** Catarina Ferreira, Santosh K. Bikkarolla, Karolin Frykholm, Saga Pohjanen, Margarida Brito, Catarina Lameiras, Olga C. Nunes, Fredrik Westerlund, Célia M. Manaia

**Affiliations:** 1 CBQF - Centro de Biotecnologia e Química Fina – Laboratório Associado, Escola Superior de Biotecnologia, Universidade Católica Portuguesa, Porto, Portugal; 2 Division of Chemical Biology, Department of Biology and Biological Engineering, Chalmers University of Technology, Gothenburg, Sweden; 3 Serviço de Virologia IPOP-FG, Porto, Portugal; 4 Serviço de Microbiologia IPOP-FG, Porto, Portugal; 5 LEPABE - Laboratory for Process Engineering, Environment, Biotechnology and Energy, Faculty of Engineering, University of Porto, Porto, Portugal; Northwestern University Feinberg School of Medicine, UNITED STATES

## Abstract

Carbapenem-resistant *Klebsiella pneumoniae* are a major global threat in healthcare facilities. The propagation of carbapenem resistance determinants can occur through vertical transmission, with genetic elements being transmitted by the host bacterium, or by horizontal transmission, with the same genetic elements being transferred among distinct bacterial hosts. This work aimed to track carbapenem resistance transmission by *K*. *pneumoniae* in a healthcare facility. The study involved a polyphasic approach based on conjugation assays, resistance phenotype and genotype analyses, whole genome sequencing, and plasmid characterization by pulsed field gel electrophoresis and optical DNA mapping. Out of 40 *K*. *pneumoniae* clinical isolates recovered over two years, five were carbapenem- and multidrug-resistant and belonged to multilocus sequence type ST147. These isolates harboured the carbapenemase encoding *bla*_KPC-3_ gene, integrated in conjugative plasmids of 140 kbp or 55 kbp, belonging to replicon types incFIA/incFIIK or incN/incFIIK, respectively. The two distinct plasmids encoding the *bla*_KPC-3_ gene were associated with distinct genetic lineages, as confirmed by optical DNA mapping and whole genome sequence analyses. These results suggested vertical (bacterial strain-based) transmission of the carbapenem-resistance genetic elements. Determination of the mode of transmission of antibiotic resistance in healthcare facilities, only possible based on polyphasic approaches as described here, is essential to control resistance propagation.

## Introduction

The discovery of antibiotics during the 20^th^ century led to a decrease in deaths due to infectious diseases. However, today antibiotic resistance is considered a worldwide crisis, with an increasing number of infections caused by multidrug-resistant bacteria [1, 2]. Carbapenem-resistant Gram-negative bacteria, in particular *Enterobacteriaceae*, are a major cause of healthcare associated infections and increasingly disseminated in the environment [3–5]. The species *Klebsiella pneumoniae (K*. *pneumoniae)* comprises the most ubiquitous and threatening carbapenem-resistant enterobacteria, with high incidence in hospital-acquired urinary tract infections, pneumonia, septicaemia and soft tissue infections [6–8]. The highly dynamic genome of *K*. *pneumoniae* explains the frequent acquisition of resistance to multiple antibiotics, often through conjugative horizontal gene transfer involving plasmids with mobilization gene cassettes containing antibiotic resistance genes [9, 10]. Due to this ability, *K*. *pneumoniae* is frequently pioneering acquisition of antibiotic resistance, putting this species in the front line of resistance against what at a given moment is considered a last resort drug, as in the cases of last generation cephalosporins, carbapenems or colistin [11, 12]. Moreover, *K*. *pneumoniae* has been considered a primary reservoir of emerging antibiotic resistance genes. A good example is the plasmid-associated *bla*_KPC_ genes, encoding carbapenem resistance, that have rapidly spread among *Enterobacteriaceae* [8, 13]. The mosaic plasmid structure, often modified by genetic recombination events, brings important challenges to most plasmid epidemiology studies. Nonetheless, the possibility of tracking plasmids occurring in different bacterial hosts, detected in different patients or environmental niches, may have an important added value to unveil the major paths of antibiotic-resistance propagation.

Techniques frequently used to characterize plasmids include Pulsed-Field Gel Electrophoresis in combination with S1 nuclease (S1-PFGE), and PCR-based replicon typing [14]. Furthermore, high throughput DNA sequencing is increasingly used for plasmid identification and characterization. However, the successful use of such an approach relies on the comparison with already known plasmids available in public databases, requires a complicated assembly process demanding advanced bioinformatics efforts, and is an overall time-consuming process. In addition, the dynamic nature of plasmidomes, including repetitive sequences, presence of multiple replicons on a single plasmid, and presence of multiple plasmids of similar or different sizes in a cell, provide challenges to all of the mentioned techniques and might contribute to confusing or inconclusive results, giving room for the development of novel plasmid characterization methods. Optical DNA mapping (ODM), based on direct visualization of individual intact plasmid molecules using fluorescence microscopy, has shown great potential as a rapid and simple method to analyse and compare plasmids [15–18]. In ODM, large DNA molecules are labelled in a sequence specific fashion and upon stretching in nanofluidic channels and imaging by fluorescence microscopy [19], a pattern reflecting the underlying DNA sequence, a barcode, is obtained [20, 21]. The method can distinguish different plasmids in a sample, based on their sizes and barcodes, which can be used for identification [17] and tracing [15, 16, 18], for example during a resistance outbreak. Furthermore, using the CRISPR/Cas9 system makes it possible to, in the same experiment, identify a specific (resistance) gene and determine its location along the plasmid sequence [22]. Thus, detailed information that conventionally would require several different techniques to extract, can be obtained in a single, rapid experiment. Moreover, the direct inspection of plasmids offers the possibility of investigating plasmid structure rearrangements, such as insertions, deletions or co-integrate formation, features that may not be easily detected by DNA sequencing techniques, even when long read techniques are used.

In the present study, we were interested in assessing the transmission mode of plasmids, vertical or horizontal, involved in *K*. *pneumoniae* carbapenem resistance dissemination in a hospital setting. A group of five carbapenem-resistant and one carbapenem-susceptible *K*. *pneumoniae* isolates were selected from a larger data set of *K*. *pneumoniae* isolates collected from 2014 to 2016 during routine screening in a healthcare facility in Porto, Portugal. The wild isolates and the respective transconjugants were characterized by phenotypic and genotypic analyses, including ODM analysis and whole genome sequencing. ODM assays proved to be particularly useful for the identification of plasmids in transconjugants and for characterizing co-integrates. Two presumable bacterial clones, harbouring two distinct conjugative plasmids harbouring the *bla*_KPC-3_ gene, could be identified in two distinct years, suggesting vertical (bacterial) transmission in the healthcare facility.

## Material and methods

### Isolates and general characterization

All methods were carried out in accordance with relevant guidelines and regulations. The study was approved by an ethics committee at the Portuguese Institute of Oncology in Porto (CES 148/020). Clinical samples, including urine, faeces, blood and haemoculture samples were processed at the Portuguese Institute of Oncology in accordance with the manual for good laboratory practices implemented in this health unit. There was no treatment of personal data and the biological samples are not related to any data that allows the identification of individuals and the ethics committee at the Portuguese Institute of Oncology in Porto therefore waived the need for consent from the patients.

Five carbapenem-resistant *K*. *pneumoniae* isolates were selected from a group of more than 40 multidrug-resistant isolates from urine, faeces or blood samples of hospitalized patients, collected over a period of 18 months, from 2014 to 2016 in Porto, Portugal. In addition, one carbapenem-susceptible clinical isolate was included for further epidemiology assessment. Isolates KP1-349 (faeces), KP1-080 (haemoculture), and KP1-388 (urine, isolated 45 days after the first two) were recovered from patient 1 in 2016 and KP2-448 and KP2-465 (urine, isolated with an interval of 15 days) from patient 2 in 2015. The selection was based on the fact that these isolates were carbapenem-resistant, later observed to be due to a *bla*_KPC-3_ gene, which might be transferred by conjugation. Isolate KP3-685 (pleural fluid) was recovered from patient 3 in 2014, at least five months before the others. This isolate, carbapenem-susceptible and lacking the *bla*_KPC-3_ gene, contained a 150 kbp plasmid that was hypothesized to be a potential ancestor of the *bla*_KPC-3_ gene carrying plasmids, and the isolate was analysed to test this hypothesis.

The identification of the isolates and the antibiotic susceptibility tests were conducted using the MicroScan WalkAway (Beckman Coulter), VITEK 2 Compact Systems (bioMérieux) and disk diffusion method or E-test (MIC Evaluator, Oxoid), respectively. Antibiotic resistance genes and plasmid replicon types were screened by PCR. The detection of β-lactamase encoding genes *bla*_TEM_, *bla*_OXA-1_, *bla*_CTX-M_ and *bla*_SHV_, and plasmid replicon types was performed as previously described [23]. The presence of carbapenemase encoding genes *bla*_KPC_, *bla*_VIM_ and *bla*_IMP_ was screened as described by Gootz *et al*., [24] Bisiklis *et al*., [25] and Mendes *et al*., [26] respectively. The colistin resistance genes *mcr-1 and mcr-2* were screened for as described by Liu *et al*., [27] and Xavier *et al*., [28] respectively.

Plasmid number and size, in both wild and transconjugant isolates, were determined by Pulsed-Field Gel Electrophoresis (PFGE), as previously described [29] and ODM with identification of the *bla*_KPC-3_ gene on plasmids linearized by CRISPR/Cas9 [22]. The genomes of the wild carbapenem-resistant *K*. *pneumoniae* isolates were characterized based on whole genome sequencing. Two wastewater *K*. *pneumoniae* isolates, strain SCC.99 and E2FC13 [30, 31] were used as recipients in conjugation assays.

### DNA extraction and sequencing

For DNA extraction, the QIAGEN Plasmid Midi Kit (QIAGEN, Germany) was used according to the manufacturer’s instructions for low-copy plasmids. Whole genome sequencing of carbapenem-resistant isolates was based on the Illumina HiSeq method with a read length of 2 x 150 bp. The following raw reads were obtained per isolate: 15 028 352 for KP1-388, 19 221 864 for KP1-080, 13 676 708 for KP1-349, 16 325 574 for KP2-448 and 12 421 310 for isolate KP2-465. The quality of the raw reads was checked with FastQC software v0.11.18, the genomes were assembled using SPAdes v3.11.1 and after the assembly the contigs with low coverage or with a size below 500 bp were removed. The putative plasmid DNA sequences were extracted using *de novo* assembled raw reads with plasmidSPAdes v3.11.1. The Average Nucleotide Identity (ANI) values between the draft genome and putative plasmid sequences of the five isolates were calculated using ANI calculator available at http://enve-omics.ce.gatech.edu/ani. Whole genome sequences were screened to determine: i) the Multilocus Sequence Type (MLST) through the web-based tool MLST [32]; ii) plasmid replicon type, identified using the plasmidFinder tool [33]; iii)) antibiotic resistance genes with the ResFinder tool [34] and the read mapping-based tool SRST2, which uses the CARD database for the detection of acquired resistance genes [35]. The search for the mobile gene elements holding the *bla*_KPC-3_ gene was performed using *de novo* assembled raw reads with the plasmidSPAdes v3.11.1 software. Plasmid assembly assays used either the total number of reads (min 12 million reads; max 19 million reads) or 3.5 million reads, aiming at reducing the number of contigs. For further analysis, the contigs originating from using 3.5 million reads were used. The gene prediction and annotation were based on the RAST server query (http://rast.nmpdr.org/rast.cgi). The obtained contigs were screened for the transposon Tn*4401*, often associated with the *bla*_KPC-3_ gene, using the TETyper and default parameters [36].

Sequencing data of the carbapenem-resistant *K*. *pneumoniae* isolates were deposited at Sequence Read Archive (SRA) accession number PRJNA588474.

### Conjugation assays

Wild carbapenem-resistant *K*. *pneumoniae* isolates were tested as donors in conjugation assays with wastewater isolates (SCC.99 and E2FC13) of the same species as recipients. Strain SCC.99 harboured plasmids of 170 kbp, 70 kbp and 30 kbp, and strain E2FC13 harboured a plasmid of 200 kbp. Conjugation assays were performed as previously described [37]. Briefly, 30°C overnight Luria-Bertani (LB) cultures of donor and recipient cells were mixed in a proportion of 1:3 (300 μL of donor cells and 900 μL of recipient cells), centrifuged for 5 min at 10 000 rpm and suspended in a final volume of 600 μL of LB medium. Potential transconjugants were isolated, after 8 hours incubation at 28 °C, on Luria-Bertani agar (LA) supplemented with tetracycline (16 mg/L), for recipient selection, and meropenem (0.25 mg/L) for transconjugant selection. Transconjugants were further characterized by antibiotic resistance phenotype and genotype analyses and ODM.

### Optical DNA mapping

The plasmid DNA of the wild carbapenem-resistant *K*. *pneumoniae* isolates, KP1-388, KP1-080, KP1-349, KP2-448, KP2-465, the respective transconjugants and the clinical isolate KP3-685 was extracted as described above. To detect the carbapenem resistance *bla*_KPC-3_ gene, plasmids were linearized by Cas9 cutting, as described elsewhere [22]. TracrRNA and crRNA with a sequence identifying the *bla*_KPC-3_ gene (5’ CAACCACCGCATCCGCGCGG 3’) were purchased from GE Healthcare (USA) and re-suspended in 10 mM RNase free Tris- HCl buffer (Sigma-Aldrich, USA). Guide RNA (gRNA) was created by incubating 0.5 nmol tracrRNA with 0.5 nmol crRNA in 1 X NEBuffer 3 (New England Biolabs, USA) and 1 X (0.1 μg/μL) BSA (New England Biolabs, USA) for 30 min at 4°C. Next, 10 μM (0.05 nmol) of gRNA was incubated with 600 ng of Cas9 (PNA Bio Inc., USA), 1 X NEBuffer 3 and 1 X (0.1 μg/μL) BSA, at 37°C for 15 min. Finally, 60 ng of plasmid DNA from the clinical isolates or the transconjugants, respectively, was added to the mixture, followed by incubation at 37°C for 1 hour.

Following the Cas9 restriction, the plasmid DNA was stained according to a competitive binding assay [20, 21] to obtain the sequence-specific pattern of the optical map. Plasmid DNA (including the Cas9 mixture) was mixed with YOYO-1 (Invitrogen, USA) and netropsin (Sigma-Aldrich, USA) at molar ratios of 2:1:100 (DNA bp:YOYO-1:netropsin). λ-DNA (48 502 bp, Roche, Switzerland) was included in the sample as an internal size reference. Samples were mixed in 5 X TBE buffer (Tris-Borate-EDTA, Novex, Invitrogen, USA) and incubated at 50°C for 30–60 min to accelerate the equilibration of YOYO-1 binding to DNA [38] and subsequently diluted with mQ-water to a final buffer concentration of 0.05 X TBE and 0.15 μM (basepairs) DNA (0.05 μM plasmid DNA + 0.1 μM λ-DNA). β-mercaptoethanol (Sigma-Aldrich, USA) was added to the sample at 2.5% (v/v) as an oxygen scavenger to reduce photonicking during visualization.

The plasmid molecules were stretched in nanofluidic channels and the optical maps were visualized using fluorescence microscopy as previously described [29]. Series of images of both circular and linear DNA molecules were obtained and used to measure the plasmid size, visualize the sequence specific pattern, and detect the presence of the *bla*_KPC-3_ gene. Images were analysed using an automated, custom-written MatLab-based program as previously described [22]. In short, the extensions of the DNA molecules were extracted from the microscopy image stacks and the molecules were clustered based on their size. For a given cluster, the associated DNA optical maps, or barcodes, were aligned and averaged to create so called consensus barcodes. By analysing whether the plasmids were linearized at random positions (due to mechanical forces or photonicking), or whether all double strand breaks occurred at the same position (cut by Cas9), the presence of *bla*_KPC-3_ was verified and its location along the plasmid sequence-based barcode was identified from the optical map. Finally, the consensus barcodes of plasmids from different samples, i.e. the clinical isolates and transconjugants, were compared [18].

The data obtained from the ODM analysis has been uploaded and is available at https://doi.org/10.5281/zenodo.4442070.

## Results

The aims of the study were to localize the carbapenem resistance gene in plasmids, assess if these plasmids were conjugative and infer if carbapenem-resistance was being transferred vertically or horizontally in this hospital. Plasmid and bacterial host characterization were combined in order to assess vertical or horizontal transmission of the carbapenemase encoding gene.

### Genomic characterization of wild isolates

From a group of 40 *K*. *pneumoniae* isolates recovered from patients over a two-year period in the studied hospital, five were carbapenem-resistant (KP1-388, KP2-448, KP2-465, KP1-080 and KP1-349), and were isolated from distinct biological samples (faeces, urine and blood) of two patients, at different time points over a period of 18 months. These isolates were *bla*_KPC_-positive, presented multidrug resistance profiles to β-lactams, aminoglycosides, quinolones, cephalosporins, sulphonamides and carbapenems, and susceptibility to tetracycline (Table 1). Although the five isolates belonged to the same MLST group, ST147 (Table 1), based on whole genome sequence analysis, they could be divided into two groups, one comprising the patient 1 isolates (KP1-388, KP1-080 and KP1-349) and the other the patient 2 isolates (KP2-448 and KP2-465). Within each group an Average Nucleotide Identity (ANI) value of 100%, and ≥99.97% for putative plasmids, was observed (S1 Table). The two groups shared ANI values ≥ 99.96%, being ≥ 96.67% for putative plasmid sequences. These results suggest that the two groups of isolates represent two distinct clones, one isolated from patient 1 during 2016 and the other from patient 2 in 2015. The isolates from both patients also differed in the plasmid replicon types. The isolates from patient 1 (n = 3) had the incFIIK plasmid replicon type combined with incFIA, whereas the isolates from patient 2 (n = 2) had incFIIK combined with incN and incFIBK replicon types (Table 2). The plasmid replicon type incN was detected by both PCR and genome sequence analysis and replicon type incFIBK was detected only by genome sequence analysis.

**Table 1:** Phenotypic and genotypic characteristics of carbapenem-resistant clinical isolates and transconjugants of *Klebsiella pneumoniae*, as well as of the wastewater *Klebsiella pneumoniae* isolates used as recipient cells in the conjugation assays.

Isolates and transconjugants									Antibiotic resistance genes			Antibiotic resistance phenotype														
	Origin	Sequence Type	Isolation Year	Plasmids PFGE (~kbp)	PCR-plasmid replicon type	MIC_GEN_ (μg/ml)	MIC_CAZ_ (μg/ml)	MIC_MEM_ (μg/ml)	β-lactamase			AMC	AML	TIC	ATM	MEM	CTX	CP	AK	STR	CIP	SXT	SUL	W	CT	TET
**Wild-type isolates**																										
**KP1-388**	**Urine**	**ST147**	**2016**	**290/140**	**FIA/FIIK**	**6**	**32**	**3**	***bla***_**TEM-1**_	***-***	***bla***_**KPC-3**_	**R**	**R**	**R**	**R**	**R**	**R**	**R**	**I**	**R**	**R**	**R**	**R**	**R**	**R**	**S**
**KP1-080**	**Haemoculture**	**ST147**	**2016**	**290/140**	**FIA/FIIK**	**16**	**128**	**2**	***bla***_**TEM-1**_	***-***	***bla***_**KPC-3**_	**R**	**R**	**R**	**R**	**R**	**R**	**R**	**I**	**R**	**R**	**R**	**R**	**R**	**S**	**S**
**KP1-349**	**Faeces**	**ST147**	**2016**	**290/140**	**FIA/FIIK**	**16**	**32**	**1.5**	***bla***_**TEM-1**_	***-***	***bla***_**KPC-3**_	**R**	**R**	**R**	**R**	**R**	**R**	**R**	**R**	**R**	**R**	**R**	**R**	**R**	**S**	**S**
**KP2-448**	**Urine**	**ST147**	**2015**	**160/136/110/55**	**N/FIIK**	**256**	**32**	**1**	***-***	***bla***_**OXA-1**_	***bla***_**KPC-3**_	**R**	**R**	**R**	**R**	**R**	**R**	**R**	**I**	**R**	**R**	**R**	**R**	**R**	**S**	**S**
**KP2-465**	**Urine**	**ST147**	**2015**	**150/136/110/55**	**N/FIIK**	**64**	**48**	**1**	***-***	***bla***_**OXA-1**_	***bla***_**KPC-3**_	**R**	**R**	**R**	**R**	**R**	**R**	**R**	**S**	**I**	**R**	**R**	**R**	**R**	**S**	**S**
**KP3-685**	**Pleural fluid**	**ND**	**2014**	**150/80**	**incF/FIIK**	**ND**	**ND**	**0.015**	***-***	***bla***_**OXA-1**_	***-***	**ND**	**R**	**R**	**ND**	**S**	**ND**	**S**	**ND**	**R**	**S**	**R**	**R**	**ND**	**S**	**S**
**Transconjugants**																										
**KP1-388_SCC.99 (E5)**				**170/140/70/30**	**FIA/FIIK**	**32**	**80**	**1**	***bla***_**TEM-1**_	***bla***_**OXA-1**_	***bla***_**KPC-3**_	**R**	**R**	**R**	**R**	**R**	**R**	**R**	**I**	**R**	**R**	**R**	**R**	**R**	**S**	**R**
**KP1-388_E2FC13 (F10)**				**200/140**	**FIA/FIIK**	**16**	**32**	**1.5**	***bla***_**TEM-1**_	**-**	***bla***_**KPC-3**_	**R**	**R**	**R**	**R**	**R**	**R**	**R**	**I**	**R**	**S**	**R**	**R**	**R**	**S**	**R**
**KP1-080_E2FC13 (C1)**				**200/140**	**FIA/FIIK**	**16**	**40**	**2**	***bla***_**TEM-1**_	**-**	***bla***_**KPC-3**_	**R**	**R**	**R**	**R**	**R**	**R**	**R**	**I**	**R**	**S**	**R**	**R**	**R**	**S**	**R**
**KP1-349_E2FC13 (A1)**				**200/140**	**FIA/FIIK**	**12**	**24**	**1**	***bla***_**TEM-1**_	**-**	***bla***_**KPC-3**_	**R**	**R**	**R**	**R**	**R**	**R**	**R**	**I**	**R**	**S**	**R**	**R**	**R**	**S**	**R**
**KP2-448_E2FC13 (B3)**				**200/55**	**N/FIIK**	**0.5**	**64**	**2**	**-**	**-**	***bla***_**KPC-3**_	**R**	**R**	**R**	**R**	**R**	**R**	**R**	**S**	**I**	**S**	**S**	**R**	**S**	**S**	**R**
**KP2-448_E2FC13 (B4)**				**200/170**	**N/FIIK**	**128**	**48**	**2**	**-**	***-***	***bla***_**KPC-3**_	**R**	**R**	**R**	**R**	**R**	**R**	**R**	**S**	**I**	**S**	**S**	**R**	**S**	**S**	**R**
**KP2-465_E2FC13 (D2)**				**200/55**	**N/FIIK**	**0,5**	**48**	**3**	**-**	**-**	***bla***_**KPC-3**_	**R**	**R**	**R**	**R**	**R**	**R**	**R**	**S**	**R**	**I**	**S**	**R**	**R**	**S**	**R**
**Recipients**																										
**SCC.99**	**Wastewater**			**170/70/30**	**FIIK**	**24**	**24**	**0,015**	**-**	***bla***_***OXA-1***_	**-**	**R**	**R**	**R**	**R**	**S**	**R**	**R**	**S**	**S**	**R**	**R**	**R**	**R**	**S**	**R**
**E2FC13**	**Wastewater**			**200**	**FIIK**	**0.5**	**0.094**	**0.015**	**-**	**-**	**-**	**R**	**R**	**R**	**R**	**S**	**R**	**S**	**S**	**I**	**S**	**S**	**R**	**R**	**S**	**R**

None of the clinical isolates harboured the antibiotic resistance genes *bla*_VIM_ and *bla*_IMP_. The KP1-388 isolate resistant to colistin lacked the *mcr-1* and *mcr-2* genes. The environmental *Klebsiella pneumoniae* SCC.99 and E2FC13 strains harboured the *bla*_CTX-M_ and *bla*_SHV_ genes. The antibiotics tested according to the standard disc diffusion method were: β-lactams (Amoxicillin+Clavulanate, AMC 20/10 μg; Amoxicillin, AML 25 μg; Ticarcillin, TIC, 75 μg; Aztreonam, ATM 30 μg; Meropenem, MEM 10 μg; Cefotaxime, CTX 30 μg; Cephalothin, CP, 30 μg), aminoglycosides (Amikacin, AK 30 μg; Streptomycin, STR, 10 μg), quinolones (Ciprofloxacin, CIP 5 μg), sulfonamides (Sulfamethoxazole/Trimethoprim, SXT 1.25/23.75 μg; Sulfamethoxazole, SUL 25 μg; Trimethoprim, W 5 μg) and tetracyclines (Tetracycline, TET 30 μg). The susceptibility to the lipopeptide, colistin (CT) was tested by the reference method, Vitek 2^®^ automated system (bioMérieux). Minimum inhibitory concentration for gentamicin (MIC_GEN_), meropenem (MIC_MEM_) and ceftazidime (MIC_CAZ_) were determined according to the E-test (MIC Evaluator, Oxoid). -, not detected; ND, not determined.

**Table 2:** Genetic determinants associated with resistance to different classes of antibiotics found in each clinical *Klebsiella pneumoniae* isolate resistant to meropenem based on whole genome sequence.

Isolates	Aminoglycoside							β -lactam					Fluoroquinolone			Fosfomycin	Phenicol	Rifampicin	Sulphonamide		Trimethoprim			Streptomycin	Plasmid Replicon types			
**KP1-388**		*aac(6’)-Ib*		*aph(3’’)-Ib*	*aph(6)-Id*	*aadA1*		bla_KPC-3_		*bla*_OXA-9_	*bla*_SHV-11_	*bla*_TEM-1_	*oqxA*	*oqxB*		*fosA*				*sul2*		*dfrA14*		*strA/strB*	FIA		FIIK	
**KP1-080**		*aac(6’)-Ib*		*aph(3’’)-Ib*	*aph(6)-Id*	*aadA1*		bla_KPC-3_		*bla*_OXA-9_	*bla*_SHV-11_	*bla*_TEM-1_	*oqxA*	*oqxB*		*fosA*				*sul2*		*dfrA14*		*strA/strB*	FIA		FIIK	
**KP1-349**		*aac(6’)-Ib*		*aph(3’’)-Ib*	*aph(6)-Id*	*aadA1*		*bla*_KPC-3_		*bla*_OXA-9_	*bla*_SHV-11_	*bla*_TEM-1_	*oqxA*	*oqxB*		*fosA*				*sul2*		*dfrA14*		*strA/strB*	FIA		FIIK	
**KP2-448**	*aac(3)-IIa*		*aac(6’)-Ib-cr*			*aadA1*	*aadA16*	*bla*_KPC-3_	*bla*_OXA-1_		*bla*_SHV-1_		*oqxA*	*oqxB*	*qnrB6*	*fosA*	*catB4*	*ARR-3*	*sul1*		*dfrA1*		*dfrA27*			FIBK	FIIK	N
**KP2-465**	*aac(3)-IIa*		*aac(6’)-Ib-cr*				*aadA16*	*bla*_KPC-3_	*bla*_OXA-1_		*bla*_SHV-11_		*oqxA*	*oqxB*	*qnrB6*	*fosA*	*catB4*	*ARR-3*	*sul1*				*dfrA27*			FIBK	FIIK	N

The antibiotic resistance genes were identified using the web-based tool Resfinder and the script SRST2 containing the databases of CARD.

**Fig 1:**
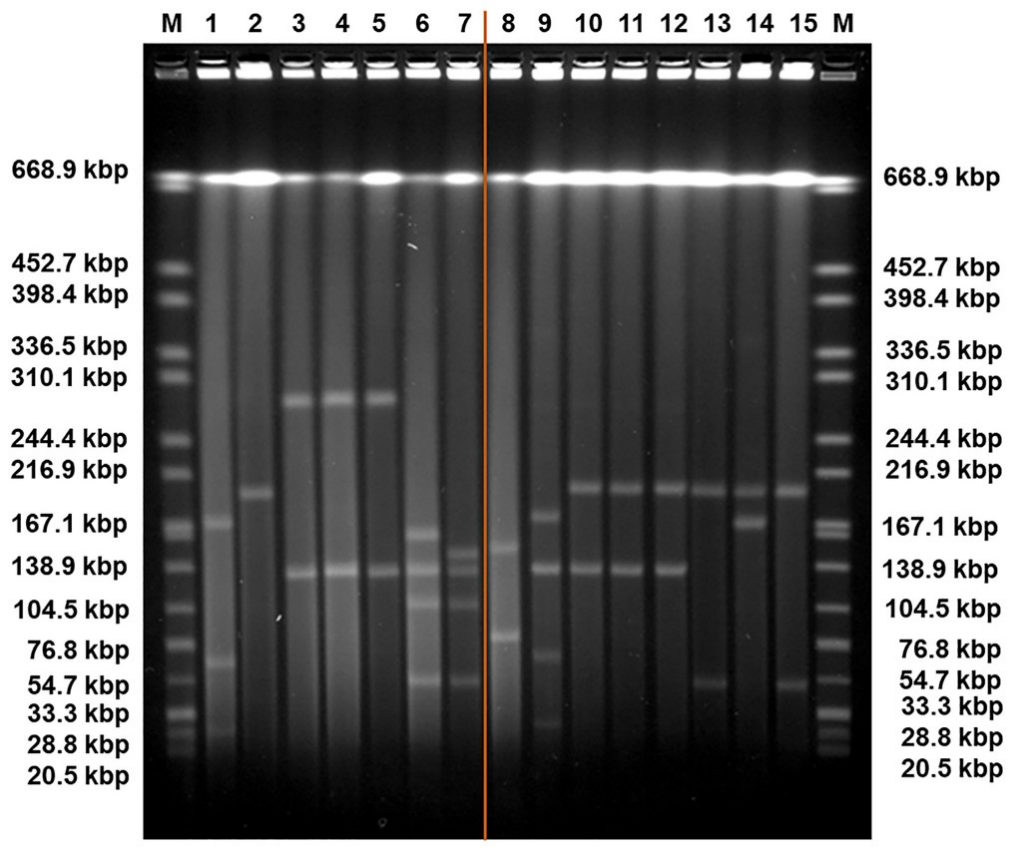
Pulse-Field Gel Electrophoresis (PFGE) of the plasmids of *Klebsiella pneumoniae* isolates SC.99 (1), E2FC13 (2), KP1-388 (3), KP1-080 (4), KP1-349 (5), KP2-448 (6), KP2-465 (7), KP3-685 (8), and the plasmids of the transconjugants E5: KP1-388-SCC.99 (9), F10: KP1-388-E2FC13 (10), C1: KP1-080-E2FC13 (11), A1: KP1-349-E2FC13 (12), B3: KP2-448-E2FC13 (13), B4: KP2-448-E2FC13 (14), D2: KP2-465-E2FC13 (15). The orange line represents a sample irrelevant for the study and cropped from the original figure, shown in S1 Fig. In conjugation assays, the strains used as recipient cells were SC.99 and E2FC13 with three plasmids (170 kbp, 70 kbp, 30 kbp) and one plasmid (200 kbp), respectively. The isolates used as donor cells were KP1-388, KP1-080 and KP1-349, all with two plasmids with same sizes (290 kbp and 140 kbp) and KP2-448 and KP2-465, each with four plasmids (160 kbp, 136 kbp, 110 kbp, 55 kbp and 150 kbp, 136 kbp, 110 kbp, 55 kbp, respectively). The isolate KP3-685 has two plasmids (150 kbp, 80 kbp). The transconjugant E5 has four plasmids (170 kbp, 140 kbp, 70 kbp, 30 kbp). The transconjugants F10, C1 and A1 have two plasmids with same sizes (200 kbp, 140 kbp). The transconjugants B3 and D2 have also the plasmid of 200 kbp and a plasmid of 55 kbp. The transconjugant B4 has the plasmid of 200 kbp and a plasmid of 170 kbp. The DNA ladder (M) contains *Salmonella enterica* serovar Braenderup H9812 (ATCC) 239 digested with XbaI.

**Fig 2:**
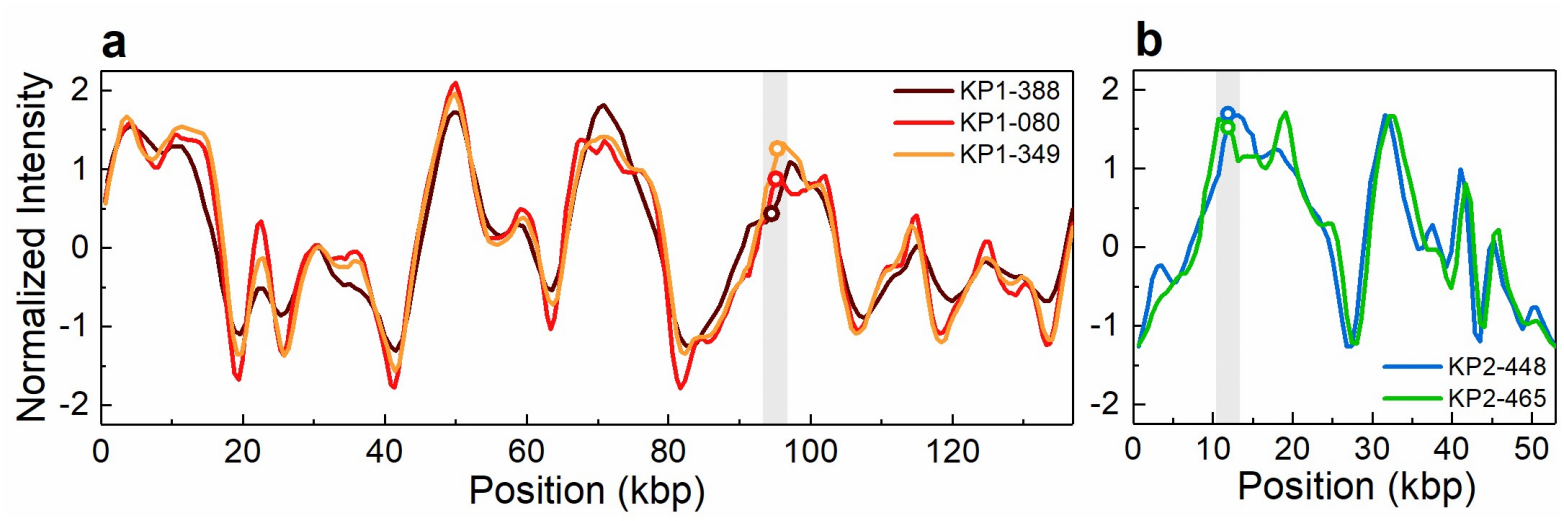
Intensity profiles reflecting the optical maps of the plasmids detected in clinical isolates and found to contain the *bla*_KPC-3_ gene. (a) The 140 kbp plasmid detected in isolates KP1-388, KP1-080 and KP1-349. (b) The 55 kbp plasmid detected in isolates KP2-448 and KP2-465. The open circles in the shaded regions indicate the location of the *bla*_KPC-3_ gene. The circle indicating the gene location in KP2-448 in (b) has been shifted vertically for increased visibility.

The antibiotic resistance gene profile, determined based on whole genome sequence analysis, confirmed that the five carbapenem-resistant isolates carried the *bla*_KPC-3_ gene variant. Moreover, this analysis revealed distinct genetic determinants in the isolates from patient 1 and patient 2, although targeting the same antibiotic classes (Table 2). Specifically, the isolates from patient 1 carried the *bla*_OXA-9_, *bla*_TEM-1_, *sul2* and *dfrA14* genes, while the isolates from patient 2 harboured the *bla*_OXA-1_, *qnr*B6, *sul1* and *dfrA27* genes. Also, the genes *catB4* and *arr-3*, encoding resistance to phenicol and rifampicin, respectively, were observed only in the genomes of the isolates from patient 2, while streptomycin resistance (*srtAB*) was found only in the isolates from patient 1. The *bla*_KPC_ genes have been described as being frequently associated with the replicative transposon Tn*4401b*, a Tn3-based transposon, flanked by two 39 bp imperfect inverted repeats, composed of two insertion sequences, IS*Kpn6* and IS*Kpn7*, a transposase gene (*tnpA*) and a resolvase gene (*tnpR*) [39]. In all isolates studied here the *bla*_KPC-3_ gene was observed to be associated with the Tn*4401d* isoform, which has a deletion of 67 bp compared to Tn*4401b* and the single nucleotide variation C8015T distinguishing *bla*_KPC-3_ from *bla*_KPC-2_. The isolates from patient 1 had the same five base-pair target site sequence flanking the left and the right direct repeats of Tn*4401d*. In the isolates from patient 2, more than one direct repeat in both flanks were observed, suggesting multiple Tn*4401* transposition events. In addition, the Tn*4401d* in the isolates from patient 1 was inserted into a Tn*1331* element composed of the *tnpA* and *tnpR* genes and the antibiotic resistance genes *aac(6´)-lb*, *aadA1*, *bla*_OXA-9_ and *bla*_TEM-1_. A similar hybrid transposon (99.99% nucleotide sequence identity) has already been described in a incFIA conjugative plasmid harbouring *bla*_KPC-3_ (pBK30661) from *K*. *pneumoniae* [40]. The Tn*4401d* and surrounding genes in the isolates from patient 2 with incFIIK/incN/incFIBK had 100% identity with a incN conjugative plasmid from *Escherichia coli* WI1 reported by Beyrouthy *et al*., [41]. The plasmid profiles, analysed by both PFGE and ODM, differed between the two groups of isolates. PFGE results revealed two plasmids, 290 kbp and 140 kbp in size, in the isolates from patient 1 and four plasmids of 150–160 kbp, 136 kbp, 110 kbp and 55 kbp in the isolates from patient 2 (Fig 1). These plasmids were also observed by ODM, except for the 290 kbp plasmid in isolate KP1-080, potentially due to its low abundance or possible fragmentation during the extraction. Given the distinctive antibiotic resistance phenotypes and genotypes observed, seemingly correlated to the plasmid replicon types, the different plasmid profiles confirmed that the *bla*_KPC-3_ gene was associated with two discrete plasmids in the isolates from patient 1 and patient 2, respectively. ODM verified the observation that the *bla*_KPC-3_ gene was harboured on the 140 kbp plasmid in the isolates from patient 1, and on the 55 kbp plasmid in the isolates from patient 2 (Fig 2). Analysis of the intensity profiles (barcodes) obtained by ODM confirmed that the same plasmid was found in each group of isolates (from patient 1 and 2, respectively). The location of the *bla*_KPC-3_ gene was determined from the linearization position along the plasmid, caused by the specific cutting by Cas9 [22] and was confirmed to be the same in the plasmids harboured by the different isolates of each group.

**Fig 3:**
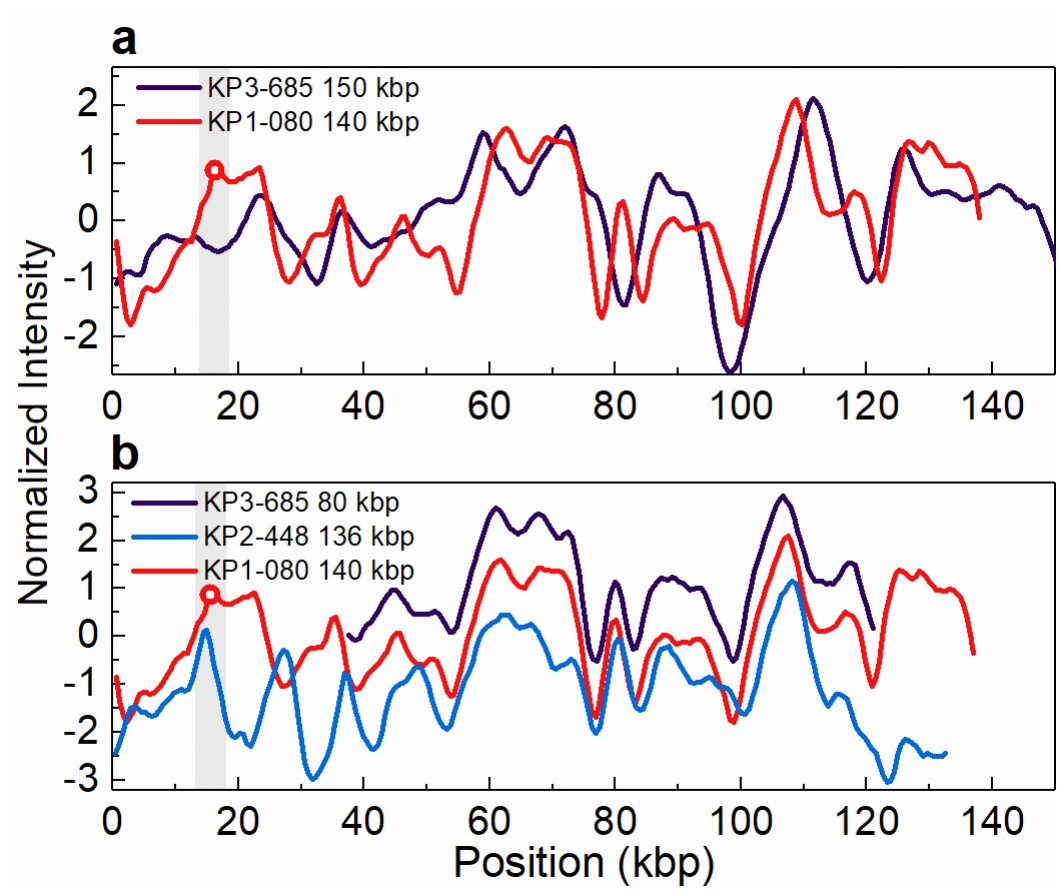
Intensity profiles reflecting the optical maps of the plasmids found in isolate KP3-685. (a) The 150 kbp plasmid present in KP3-685, lacking the *bla*_KPC-3_ gene, and the 140 kbp plasmid present in KP1-080 (patient 1), harbouring the *bla*_KPC-3_ gene (indicated by the open circle in the shaded region). Shown is the best matching comparison, with a p-value of 0.63 (threshold for similarity set to p-value ≤ 0.01). (b) The 80 kbp plasmid present in KP3-685 and the 136 kbp plasmid present in KP2-448 (patient 2), both lacking the *bla*_KPC-3_ gene, and the 140 kbp plasmid present in KP1-080 (patient 1), harbouring the *bla*_KPC-3_ gene (indicated by the open circle in the shaded region). The intensity profiles for the 80 kbp plasmid of isolate KP3-685 and 136 kbp plasmid of isolate KP2-488 have been shifted vertically (+1 and -1 unit, respectively) for increased visibility. Note that the intensity profile of the 140 kbp plasmid is shifted relative to the intensity profile shown in Fig 2, due to circular permutation.

The carbapenem-susceptible isolate KP3-685 from 2014, which lacked the *bla*_KPC-3_ gene, was found to have a plasmid with a size (~150 kbp) similar to that of the plasmid harbouring the *bla*_KPC-3_ gene in the isolates from patient 1 (Table 1, Fig 1). We suspected that this plasmid could be the genetic platform for acquisition of the *bla*_KPC-3_ gene. This hypothesis was tested using the ODM assay, but no similarity between that ~150 kbp plasmid and the *bla*_KPC-3_-harbouring 140 kbp plasmid could be found (Fig 3a). However, ODM revealed that the second plasmid, 80 kbp in size, found in isolate KP3-685 had an intensity profile that overlapped in part with the profiles of the 140 kbp plasmid observed in the isolates from patient 1 and the 136 kbp plasmid observed in the isolates from patient 2 (Table 1, Fig 3b). The 140 kbp plasmid found in the isolates from patient 1 harbours the *bla*_KPC-3_ gene, whereas neither the 136 kbp plasmid found in the isolates from patient 2 nor the 80 kbp plasmid found in isolate KP3-685 does. Consistently, the similar parts of the intensity profiles of these plasmids does not include the region where the *bla*_KPC-3_ gene is located.

### Conjugative plasmids harbouring *bla*_KPC-3_

Conjugation assays were used to further test the hypothesis that the *bla*_KPC-3_ carrying plasmids, identified by ODM, could be horizontally transferred and, in case affirmative, if they were identical to those observed in the wild isolates. In these assays, meropenem was used for selection of transconjugants.

**Fig 4:**
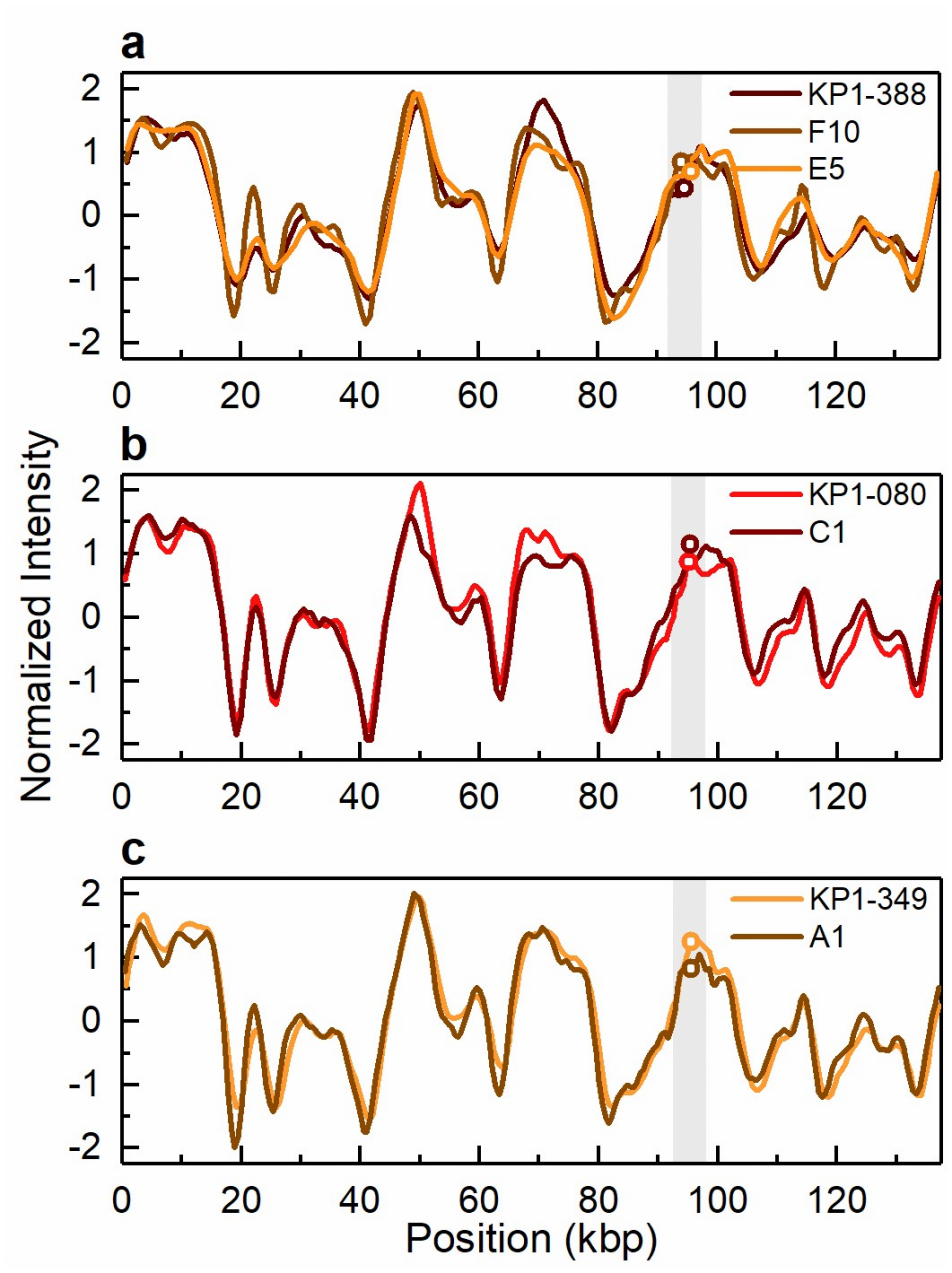
Intensity profiles reflecting the optical maps of the 140 kbp conjugative plasmid harbouring the *bla*_KPC-3_ gene. Conjugative plasmid of 140 kbp present in (a) the clinical isolate KP1-388 and the corresponding transconjugants KP1-388_E2FC13 (F10) and KP1-388_SCC.99 (E5) and (b) the clinical isolate KP1-080 and the corresponding transconjugant KP1-080_E2FC13 (C1) and (c) the clinical isolate KP1-349 and the corresponding transconjugant KP1-349_E2FC13 (A1). The open circles in the shaded regions indicate the location of the *bla*_KPC-3_ gene. The circles indicating the gene location in E5 in (a) and C1 in (b) have been shifted vertically for increased visibility.

**Fig 5:**
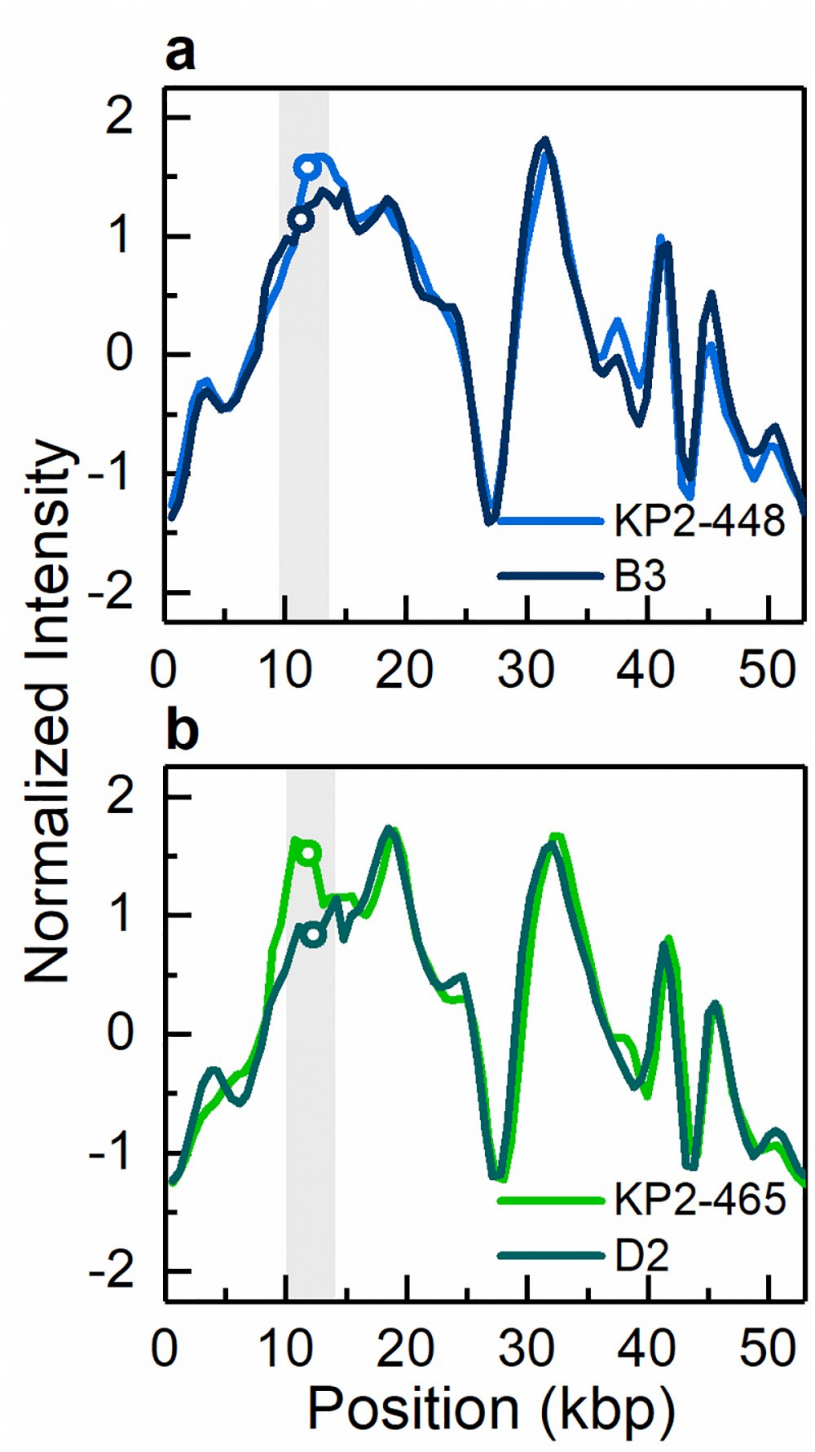
Intensity profiles reflecting the optical maps of the 55 kbp conjugative plasmid harbouring the *bla*_KPC-3_ gene. Conjugative plasmid of 55 kbp present in (a) the clinical isolate KP2-448 and the corresponding transconjugant KP2-448_E2FC13 (B3) and (b) the clinical isolate KP2-465 and the corresponding transconjugant KP2-465_E2FC13 (D2). The open circles in the shaded regions indicate the location of the *bla*_KPC-3_ gene.

**Fig 6:**
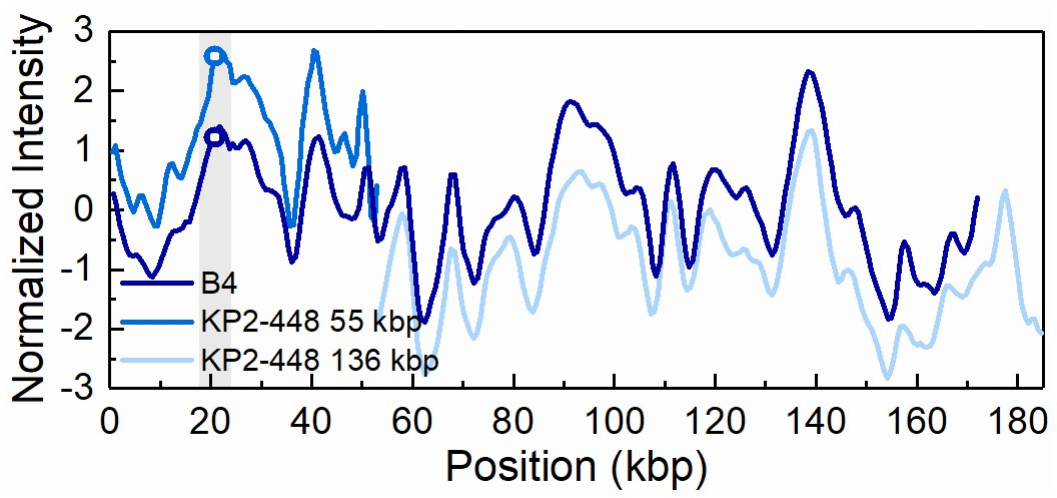
Intensity profiles reflecting the optical maps of the 170 kbp plasmid present in transconjugant KP2-448_E2FC13 (B4) and the 55 kbp and 136 kbp plasmids present in the *Klebsiella pneumoniae* clinical isolate KP2-448. The open circles in the shaded region indicate the *bla*_KPC-3_ gene position on the 55 kbp and 170 kbp plasmid, respectively. The intensity profiles for the 55 kbp and 136 kbp plasmids have been shifted vertically (+1 and -1 unit, respectively) for increased visibility.

The five carbapenem-resistant isolates were all found able to transfer the *bla*_KPC-3_ gene by conjugation. With the exception of colistin and ciprofloxacin resistance, all the tested resistance phenotypes were transferred in parallel with carbapenem resistance (Table 1). Consistently, PFGE and ODM analyses showed that transconjugants obtained from tetracycline and meropenem selection after conjugation assays of wild type isolates with the 290 kbp and 140 kbp plasmids (patient 1) received the latter plasmid, while those resulting from wild isolates with plasmids of sizes 150–160 kbp, 136 kbp, 110 kbp and 55 kbp (patient 2) received the smallest plasmid or, as observed in one case, a potential co-integrate with a novel plasmid size of 170 kbp (Figs 1 and 4–6). Based on PCR plasmid replicon typing, it was possible to conclude that the transferred 140 kbp plasmid was of plasmid replicon types incFIA/incFIIK, and the transferred 55 kbp plasmid and the 170 kbp co-integrate were of plasmid replicon types incN/incFIIK (Table 1).

ODM analysis of the transconjugant plasmids confirmed that the 140 kbp and the 55 kbp plasmids are the same as in the respective wild donors (Figs 4 and 5), without any major structural rearrangements, and that they harbour the *bla*_KPC-3_ gene. Also, the co-integrate 170 kbp plasmid present in one of the transconjugants, from isolate KP2-448, was found to harbour the *bla*_KPC-3_ gene. Based on ODM, it was possible to confirm that the 170 kbp co-integrate originate from a combination of the 55 kbp plasmid, where the *bla*_KPC-3_ gene is located, and the 136 kbp plasmid present in the wild isolate (Fig 6). Also, the location of the breakpoint in each plasmid when forming the co-integrate could be detected, and that a smaller region of the 136 kbp plasmid was lost in the co-integration process (explaining why the 170 kbp plasmid is smaller than the sum of the 55 kbp and 136 kbp plasmids). The 136 kbp plasmid present in the wild isolates from patient 2 (KP2-448 and KP2-465) was not transferred alone, but only as part of the 170 kbp co-integrate. The formation of the 170 kbp co-integrate also permitted the transfer of gentamicin resistance, presumably located on the 136 kbp plasmid of isolate KP2-448, in which a MIC_GEN_ of 256 μg/mL was determined. Indeed, in the transconjugants of this isolate, the MIC_GEN_ values raised from 0.5 μg/mL when the 55 kbp plasmid was transferred alone, to 128 μg/mL when it formed a co-integrate with the 136 kbp plasmid (Table 1).

## Discussion

Multidrug-resistant *K*. *pneumoniae* are a major clinical threat among *Enterobacteriaceae*, in terms of spread of resistance to last resort antibiotics, such as carbapenems. Horizontal gene transfer via plasmids constitutes a major pathway for the spread of antibiotic resistance and identifying and characterizing plasmids is therefore an important part of the investigation of resistant strains. In this study, we aimed to assess the diversity of carbapenem-resistant *K*. *pneumoniae* in a clinical setting and the evolution of resistance through plasmid acquisition.

Here, we show that clinical *K*. *pneumoniae* isolates, collected from two distinct patients over a period of 18 months, from the same MLST, ST147, possessed different conjugative plasmids harbouring the *bla*_KPC-3_ gene. The finding that all carbapenem-resistant isolates were from the same clonal complex is not surprising since *K*. *pneumoniae* ST147 is considered an emerging high-risk clonal lineage, primarily associated with the production of carbapenemases [42, 43]. Although belonging to the same MLST, the isolates represented two clones that harboured distinct *bla*_KPC-3_ plasmids. These observations suggest that two clones harbouring the gene *bla*_KPC-3_ colonized these patients. While no evidence of horizontal gene transfer was detected, conjugation might have taken place since both *bla*_KPC-3_ plasmids were conjugative.

The two conjugative plasmids harbouring the *bla*_KPC-3_ gene were characterized as incFIA (plasmid of size 140 kbp) and incN (plasmid of size 55 kbp), respectively. The association of *K*. *pneumoniae* ST147 with plasmids incFIA and incN has been reported before [10, 44]. The 55 kbp plasmid was in one case transferred as part of a co-integrate (of size 170 kbp), which also was characterized as incN. The formation of incN co-integrate plasmids harbouring *bla*_KPC-3_ is seldomly reported in the literature compared with the formation of incF co-integrate plasmids [40, 45]. The transconjugant carrying the 170 kbp co-integrate did not only show carbapenem resistance, but in addition a significantly higher gentamicin resistance than the transconjugant to which the 55 kbp plasmid was transferred alone (Table 1). This finding confirms the advantage of forming co-integrates in the spread of genes related to carbapenem and aminoglycoside resistance [11].

The association of carbapenem resistance genes to the Tn*4401d* transposon region was previously reported in a incN plasmid from *E*. *coli* [41]. Thus, the two features of the 55 kbp incN plasmid, that it forms co-integrate plasmids and that it shares Tn*4401d* with plasmids present in other species, shows the plasticity of this plasmid and its potential to spread antibiotic resistance among *Enterobacteriaceae*. In addition, our results show that the insertion of transposon Tn*4401*d harbouring the *bla*_KPC-3_ gene into the Tn*1331* transposon from incFIA plasmid also lead to the spread of resistance to different classes of antibiotics, as already described [40].

An important part of this study was to demonstrate the usefulness of ODM for plasmid characterization. For determination of plasmid profiles, PFGE is the most commonly used technique [14, 46]. The number of plasmids, and their sizes, in each isolate determined by ODM was consistent with the results obtained using PFGE.

The presence of antibiotic resistance genes is routinely determined by PCR-based methods and genome sequencing. Using PCR-based methods to screen for antibiotic resistance genes, or any other genes, gives the presence or absence of the given targets, whereas sequencing methods provide further information on where the gene is located, i.e. on which plasmid or on the bacterial chromosome. ODM, complemented by CRISPR/Cas9, can also be used for gene recognition and in addition reveal where along the plasmid sequence the gene is located. Thus, both of these results are obtained from one single experiment ODM, on the other hand, gives both of these results from one single experiment [22], which is unique for ODM. The ODM analysis could directly show that the gene of interest, *bla*_KPC-3_, was present on the 140 kbp plasmid in the isolates from patient 1 and on the 55 kbp plasmid in the isolates from patient 2. Moreover, while the conjugation assays showed the transfer of carbapenem resistance, ODM could confirm not only the transfer of the *bla*_KPC-3_ gene, but also the transfer of the intact plasmids carrying the resistance gene.

The transfer of the 55 kbp plasmid carrying the *bla*_KPC-3_ gene as a co-integrate, indicated by the finding of a 170 kbp plasmid in one of the carbapenem-resistant transconjugant isolates, could be verified by ODM. Moreover, not only could ODM confirm the presence of the target gene on this novel sized plasmid, but also map the origin of the co-integrate. The intensity profile of the 170 kbp co-integrate matched well with the intensity profiles of the 55 kbp plasmid carrying the *bla*_KPC-3_ gene and another plasmid of 136 kbp present in the wild isolate that did not carry the *bla*_KPC-3_ gene. Sequencing approaches could be used to confirm plasmid rearrangements, such as the formation of co-integrates, or inversions, insertions or deletions, although the dynamic and often repetitive nature of plasmid elements many times makes the assembly process of sequencing data non-trivial and time-consuming. Here, ODM serves as an advantageous alternative, as this kind of dynamic rearrangements can be directly visualized in a single, fast experiment. This study is the first example of using ODM to characterize the formation of co-integrate plasmids during conjugation.

In the carbapenem-susceptible isolate from 2014, KP3-685, two plasmids were found, of which one was of similar size as the 140 kbp plasmid harbouring the *bla*_KPC-3_ gene in the isolates from patient 1, leading to our hypothesis that the plasmids found in this isolate potentially could represent ancestors of the *bla*_KPC-3_ plasmids identified in the 2015 or 2016 isolates. The ODM analysis revealed that the plasmid with size of 80 kbp in this 2014 isolate, and not the 140 kbp plasmid, shared sequence similarities with the plasmids of around 140 kbp size observed in both patients, although not in the region where the *bla*_KPC-3_ gene was located. This could possibly be an indication of a common plasmid origin in all the investigated isolates before the acquisition of the carbapenem resistance gene, or, alternatively, the plasmid in isolate KP3-685 lost the carbapenem-resistance gene. Comparison of the intensity profiles of the two conjugative plasmids harbouring the *bla*_KPC-3_ gene (140 kbp and 55 kbp), particularly in the region where the gene is located, did however not show any significant similarity, indicating that the evolution of antibiotic resistance in these two plasmids occurred in parallel rather than from a common origin.

## Conclusions

The results of this study showed that the five carbapenem-resistant *K*. *pneumoniae* isolates recovered in the specific hospital from two patients and over a period of more than one year represented two distinct clones of the same MLST group (ST147), one with two isolates and another with three. In these two groups of isolates the *bla*_KPC-3_ gene was located on different conjugative plasmids, with a size of 140 kbp in one and 55 kbp in the other. The plasmids were different, as could be confirmed by ODM, with no hints of horizontal gene transfer of the *bla*_KPC-3_ gene. Therefore, it was suggested that the transmission of carbapenem-resistant *K*. *pneumoniae* in the hospital at the time investigated was vertical. Although high throughput sequencing is valuable for this kind of studies, ODM has unquestionable advantages if one considers the high speed and reliability versus the low cost of this method to distinguish plasmids and simultaneously identify genetic determinants. We therefore foresee that ODM can become a standard tool in plasmid analysis.

## Supporting information

S1 Fig Pulse-field Gel Electrophoresis of the plasmids of *Klebsiella pneumoniae* isolates SC.99 (1), E2FC13 (2), KP1-388 (3), KP1-080 (4), KP1-349 (5), KP2-448 (6), KP2-465 (7), KP3-685 (8), and the plasmids from transconjugants E5: KP1-388-SCC.99 (9), F10: KP1-388-E2FC13 (10), C1: KP1-080-E2FC13 (11), A1: KP1-349-E2FC13 (12), B3: KP2-448-E2FC13 (13), B4: KP2-448-E2FC13 (14), D2: KP2-465-E2FC13 (15). The sample delimited by an orange dashed line was cropped from the figure as it is a sample irrelevant for the study. In conjugation assays, the strains used as recipient cells were SC.99 and E2FC13 with three plasmids (170 kbp, 70 kbp, 30 kbp) and one plasmid (200 kbp), respectively. The isolates used as donor cells were KP1-388, KP1-080 and KP1-349, all with two plasmids with same sizes (290 kbp and 140 kbp) and KP2-448 and KP2-465, each with four plasmids (160 kbp, 136 kbp, 110 kbp, 55 kbp and 150 kbp, 136 kbp, 110 kbp, 55 kbp, respectively). The isolate KP3-685 has two plasmids (150 kbp, 80 kbp). The transconjugant E5 has four plasmids (170 kbp, 140 kbp, 70 kbp, 30 kbp). The transconjugants F10, C1 and A1 have two plasmids with same sizes (200 kbp, 140 kbp). The transconjugants B3 and D2 have also the plasmid of 200 kbp and a plasmid of 55 kbp. The transconjugant B4 has the plasmid of 200 kbp and a plasmid of 170 kbp. The DNA ladder (M) contains *Salmonella enterica* serovar Braenderup H9812 (ATCC) 239 digested with XbaI.
(DOCX)

S1 Table Similarities between draft genome comprising both putative plasmid DNA and chromosomal DNA sequences of *Klebsiella pneumoniae* isolates harbouring *bla*_KPC-3_ gene retrieved by Average Nucleotide Identity (ANI) calculator (http://enve-omics.ce.gatech.edu/ani). Values in parentheses correspond to ANI values considering putative plasmid DNA from the five isolates. Since the identity between draft genome from the five isolates were higher than 95%, all isolates are considered to belong to the same species, *Klebsiella pneumoniae*.
(DOCX)
