# Supplementary material for: Polyphasic characterization of carbapenem-resistant Klebsiella pneumoniae clinical isolates suggests vertical transmission of the blaKPC-3 gene
Source: PLoS One. 2021 Feb 26;16(2):e0247058. doi: 10.1371/journal.pone.0247058 (PMC7909683; doi:10.1371/journal.pone.0247058)
Supplement: S1 Fig — The sample delimited by an orange dashed line was cropped from the figure as it is a sample irrelevant for the study. In conjugation assays, the strains used as recipient cells were SC.99 and E2FC13 with three plasmids (170 kbp, 70 kbp, 30 kbp) and one plasmid (200 kbp), respectively. The isolates used as donor cells were KP1-388, KP1-080 and KP1-349, all with two plasmids with same sizes (290 kbp and 140 kbp) and KP2-448 and KP2-465, each with four plasmids (160 kbp, 136 kbp, 110 kbp, 55 kbp and 150 kbp, 136 kbp, 110 kbp, 55 kbp, respectively). The isolate KP3-685 has two plasmids (150 kbp, 80 kbp). The transconjugant E5 has four plasmids (170 kbp, 140 kbp, 70 kbp, 30 kbp). The transconjugants F10, C1 and A1 have two plasmids with same sizes (200 kbp, 140 kbp). The transconjugants B3 and D2 have also the plasmid of 200 kbp and a plasmid of 55 kbp. The transconjugant B4 has the plasmid of 200 kbp and a plasmid of 170 kbp. The DNA ladder (M) contains Salmonella enterica serovar Braenderup H9812 (ATCC) 239 digested with XbaI. (DOCX) [file pone.0247058.s001.docx]

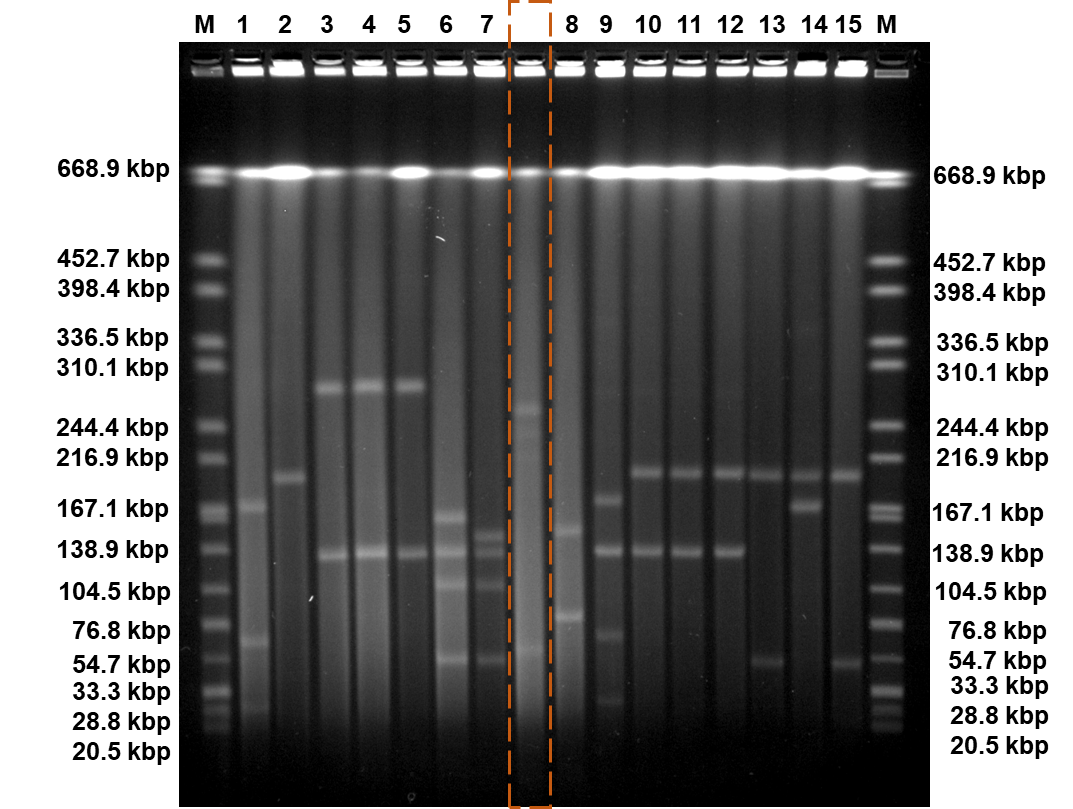


**S1 Fig.** **Pulse-field Gel Electrophoresis of the plasmids of *Klebsiella pneumoniae* isolates SC.99 (1), E2FC13 (2), KP1-388 (3), KP1-080 (4), KP1-349 (5), KP2-448 (6), KP2-465 (7), , KP3-685 (8), and the plasmids from transconjugants E5: KP1-388-SCC.99 (9), F10: KP1-388-E2FC13 (10), C1: KP1-080-E2FC13 (11), A1: KP1-349-E2FC13 (12), B3: KP2-448-E2FC13 (13), B4: KP2-448-E2FC13 (14), D2: KP2-465-E2FC13 (15).** The sample delimited by an orange dashed line was cropped from the figure as it is a sample irrelevant for the study. In conjugation assays, the strains used as recipient cells were SC.99 and E2FC13 with three plasmids (170 kbp, 70 kbp, 30 kbp) and one plasmid (200 kbp), respectively. The isolates used as donor cells were KP1-388, KP1-080 and KP1-349, all with two plasmids with same sizes (290 kbp and 140 kbp) and KP2-448 and KP2-465, each with four plasmids (160 kbp, 136 kbp, 110 kbp, 55 kbp and 150 kbp, 136 kbp, 110 kbp, 55 kbp, respectively). The isolate KP3-685 has two plasmids (150 kbp, 80 kbp). The transconjugant E5 has four plasmids (170 kbp, 140 kbp, 70 kbp, 30 kbp). The transconjugants F10, C1 and A1 have two plasmids with same sizes (200 kbp, 140 kbp). The transconjugants B3 and D2 have also the plasmid of 200 kbp and a plasmid of 55 kbp. The transconjugant B4 has the plasmid of 200 kbp and a plasmid of 170 kbp. The DNA ladder (M) contains *Salmonella enterica* serovar Braenderup H9812 (ATCC) 239 digested with XbaI.
