# Supplementary material for: Polyphasic characterization of carbapenem-resistant Klebsiella pneumoniae clinical isolates suggests vertical transmission of the blaKPC-3 gene
Source: PLoS One. 2021 Feb 26;16(2):e0247058. doi: 10.1371/journal.pone.0247058 (PMC7909683; doi:10.1371/journal.pone.0247058)
Supplement: S1 Table — Values in parentheses correspond to ANI values considering putative plasmid DNA from the five isolates. Since the identity between draft genome from the five isolates were higher than 95%, all isolates are considered to belong to the same species, Klebsiella pneumoniae. (DOCX) [file pone.0247058.s002.docx]

**S1 Table.** **Similarities between draft genome comprising both putative plasmid DNA and chromosomal DNA sequences of *Klebsiella pneumoniae* isolates harbouring *bla*_KPC-3_ gene retrieved by Average Nucleotide Identity (ANI) calculator (http://enve-omics.ce.gatech.edu/ani).** Values in parentheses correspond to ANI values considering putative plasmid DNA from the five isolates. Since the identity between draft genome from the five isolates were higher than 95%, all isolates are considered to belong to the same species, *Klebsiella pneumoniae*.

| Clinical isolates | KP1-388 | KP1-080 | KP1-349 | KP2-448 | KP2-465 |
| --- | --- | --- | --- | --- | --- |
| KP1-388 | * | 100 %  (99.98 %) | 100 %  (99.99 %) | 99.96 %  (96.85 %) | 99.96 %  (96.88 %) |
| KP1-080 | * | * | 100 %  (99.97 %) | 99.97 %  (96.67 %) | 99.97 %  (96.90 %) |
| KP1-349 | * | * | * | 99.96 %  (96.82 %) | 99.97 %  (96.85 %) |
| KP2-448 | * | * | * | * | 100 %  (99.98 %) |
| KP2-465 | * | * | * | * | * |
